# Investigating the tribological characteristics of copper surface composites reinforced with high entropy alloy (AlCoCrCuFe) through friction stir processing

**DOI:** 10.1038/s41598-023-49557-5

**Published:** 2023-12-19

**Authors:** Seenivasan Soundararajan, Gopal Pudhupalayam Muthukutti, Soorya Prakash Kumarasamy, Kavimani Vijayananth, Debabrata Barik, Prabhakar Sharma, Prabhu Paramasivam

**Affiliations:** 1 Mechanical Engineering, Rathinam Technical Campus, Coimbatore, 641021 India; 2 Center for Material Science, Mechanical Engineering, Karpagam Academy of Higher Education, Coimbatore, 641021 India; 3 Mechanical Engineering, Anna University Regional Campus, Coimbatore, 641046 India; 4 Department of Mechanical Engineering, Karpagam Academy of Higher Education, Coimbatore, 641021 India; 5 Department of Mechanical Engineering, Delhi Skill and Entrepreneurship University, New Delhi, 110089 India; 6 Department of Mechanical Engineering, College of Engineering and Technology, Mattu University, 318 Mettu, Ethiopia

**Keywords:** Corrosion, Other nanotechnology

## Abstract

The present investigation focuses on the fabrication of Copper-High Entropy Alloy (HEA) surface Metal Matrix Composite (MMC) using the solid-state Friction Stir Process (FSP) and the characterization of wear characteristics. Higher hardness values at the level of 770HV were the cornerstone in its selection, in addition to identifying several appropriate considerations for combining the AlCoCrCuFe HEA in Cu-HEA surface MMCs. Because of the combination of FSP and HEA, the produced composite had a fine microstructure and increased hardness. The wear test is carried out using pin-on-disc equipment for all conceivable parameter combinations to thoroughly analyze wear qualities, with velocity, load, as well as sliding distance chosen as input parameters. The wear rate decreases dramatically with HEA additions and rises with sliding velocity, load, and sliding distance. The impact of HEA addition on the Coefficient of Friction (CoF) during a dry sliding wear test is opposed to its influence on wear rate. The wear parameters such as load, sliding speed, and sliding distance possess a positive correlation with the wear rate and a negative correlation with a coefficient of friction. The applied load has a severe effect on wear rate and CoF when compared to other wear parameters considered. Scanning Electron Microscope (SEM) micrographs of the worn surface were utilized to analyze the wear process, which clearly showed that the copper’s wear resistance improved with the addition of HEA.

## Introduction

Copper is viewed as a material among the proficient metals utilized in industries due to elevated thermal and electrical possessions besides having superior formability and resistance to corrosion^1–3^ On the other hand, ductility, low hardness, and resistance to wear are the factors that prevent the wider application of copper^4^. Copper-based composites with ceramic reinforcements were developed to overcome these drawbacks and they become the most capable materials in many engineering fields where there is a need for higher microstructural firmness and temperature resistance^5^. Cu composites with various reinforcements were applied as brake and bearing material, superior tribe functioning dry sliding material, heat sink and spreader, and anti-corrosive coating^6–8^. It was also reported that the copper composites were utilized as brake pad materials, spacecraft docking and rendezvous, shaft bearings, and other complex high-tech domains. Even though copper composites have better properties, they also have some limitations like low strength and poor tribological properties besides having poor wettability with reinforced particles that result in lower interface strength and consequently inferior properties^9–11^.

Since the copper matrix has poor wettability with reinforced ceramics, it is advisable to find alternate compatible reinforcement to develop high-performance composites. Also, the higher difference in the thermal characteristics between matrix and ceramics makes the metal-ceramic composites fail in higher temperature applications^12^. So, a material other than ceramics that too with better properties has to be identified as an alternative reinforcement. High Entropy Alloys (HEAs) and metallic glasses were identified as potential materials of reinforcements in metal matrix composites whereas the low crystallization temperature and poor plasticity are the two major setbacks for metallic glasses to be reinforced.

HEAs are a new kind of designed alloy that consists of several components with equal atomic ratio or nearer to equal atomic ratio, quite opposite to conventional alloys where alloying elements are added in small quantities with one or two major principal elements^13,14^. High entropy, rigorous lattice alteration, indolent diffusion, and cocktail are the major effects that fallout in the formation of HEA as a simple solid solution structure with exceptional high-temperature characteristics inclusive of hardness, strength, corrosion resistance, and wear^15^. At high-temperature circumstances, some HEAs like NbTaWMo and NbTaWMoV exhibit higher strength than the Haynes 230 and Inconel 718^16^. Hence, HEAs are also utilized as a filler material in dissimilar laser welding of stainless steel and aluminum^17^. Experimental research on HEA as a reinforcing medium is in the growing stage where several researchers tried different HEAs as reinforcements and reported better properties. Analysis of Cu- AlCoNiCrFe HEA composite fabricated through powder metallurgy reveals that the composite has no evidence of unwanted grain growth and intermetallic phase formation while the yield strength of copper was found to increase with HEA addition^18^. An enhancement in hardness of 63.7% was achieved when CoCrFeMnNi was reinforced with Al 2024 alloy^19^. Utilization of Al_0.3_CoCrFeNi HEA particles as reinforcement in copper matrix resulted in an improvement of wear resistance^20^. Investigation of the effect of AlCrFeMnNi HEA on the Aluminum MMC reveals that hardness and wear resistance increase with the increase in HEA particle^21^. Fe_29_Cr_28_Mn_19_Ni_18_Al_5_Si reinforced Al MMC shows the addition of HEA in the aluminum matrix has a significant influence on friction coefficient^22^. Various studies on HEA-reinforced composites have already proven HEA as a potential reinforcement material for composite fabrication whereas its effect on wear resistance of test composite at different sliding conditions is not explored in detail.

The incorporation of hard reinforcements to the copper matrix also hurts ductility and toughness of the material^23^. Given this fact, several researchers opted to reinforce the hard materials on the surface alone instead of bulk reinforcement which is also named as surface composites. The surface composite concept helps to develop a hard, wear, and corrosion-resistant surface without any compromise in the volumetric properties of the matrix body. Friction Stir technique is one of the surface composite fabrication methodologies in which the composite fabrication is made well below the melting temperature of constituent materials^24^. It is derived from friction stir welding in which a non-consumable tool is plunged into the surface for joining^25^. FSP is a thermomechanical process derived from friction stir welding in which plastic deformation can yield an ultrafine^26–29^. FSP has gained attention from researchers and industry as it modifies the microstructure of the material, improving ductility without forfeiting strength^30^. More importantly, FSP is also a more economical and dependable technique for surface composite fabrication. From the literature review, it can be noted that the copper composite has many better properties except poor wear resistance, and copper has poor wettability with ceramic reinforcements resulting in inferior properties. So, the current research aims to develop a novel wear-resistant copper composite with an alternate compatible reinforcement material called HEA. Only minimal researchers reported on HEA-incorporated copper composites and none has analyzed their wear properties in detail. Since the wear resistance is a surface property of the material, it is planned to reinforce the novel HEA reinforcement in copper surface alone through FSP methodology. Utilization of FSP methodology aids in enhancing the surface characteristics of the copper without affecting its bulk properties. Hence it is proposed to develop a wear-resistant Cu-HEA composite through the FSP technique and analyze its wear properties in detail.

## Experimental procedure

### Composite preparation

**Figure 1:**
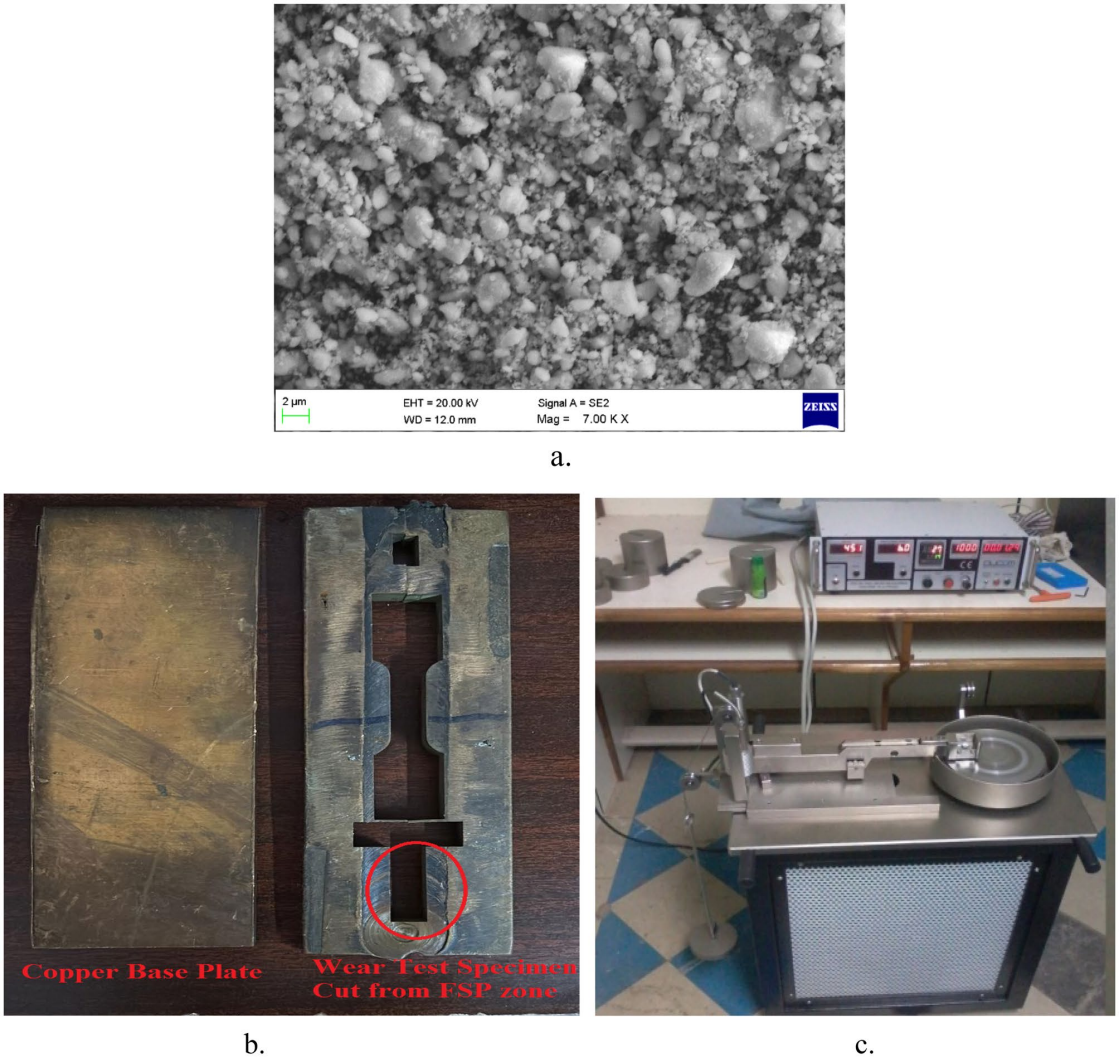
(**a**) HEA reinforcement, (**b**) copper base material and wear test specimen, (**c**) pin on disc wear test.

Copperplate of 150 × 75 × 8 mm dimensions (Fig. 1a) is taken as base material whereas AlCoCrCuFe HEA particles are chosen as reinforcing medium. The selection of the AlCoCrCuFe high-entropy alloy (HEA) as a reinforcement material is primarily driven by its notable achievement of possessing a high hardness value of approximately 770HV. AlCoCrCuFe HEA is developed through the arc melting method in which a measured quantity of high-purity initial materials Al, Co, Cr, Cu, and Fe is fed into a graphite crucible, and the high-energy arc is supplied. Since the developed HEA is equiatomic, the initial materials were taken in equal atomic % as given in Table 1. High energy arc melts the materials HEA is formed and the developed HEA is converted into fine particles with the aid of ball milling. The morphology of the prepared HEA reinforcement is provided in Fig. 1a. Surface composite development through FSP involves three main processes, (a) groove making, (b) compacting with reinforcement particles, and (c) traversing of tools (with and without pin)^31^. A groove was machined on the surface of the copper plate to fill the HEA particle for mixing in the base plate during friction stir processing. The groove was machined using a wire electrical discharge machine. The dimensions of the machined groove are 150 mm in length and 5 mm in depth. The width of the groove was varied according to the volume percentage of HEA particles to be reinforced. The present study is aimed to develop a surface composite with 5, 10, and 15 vol% of HEA particles. According to this, the groove width is machined for 0.3, 0.6, and 0.9 mm respectively. The volume fraction of the composite is calculated using the following expressions.

1 $$Vf = \frac{A}{Ap}$$

2 $$A = W \times T$$

3 $$Ap = D \times L$$

where, *Vf*—volume fraction, A—area of the groove, Ap—projected area of tool pin, W—width of the groove, T—depth of the groove, D—diameter of the pin and L—length of the pin.

**Table 1:** HEA composition.

Material	Al	Co	Cr	Cu	Fe
Atomic %	20	20	20	20	20

HEA particles were filled in the machined groove on a copper plate which is then clamped rigidly in an indigenously built FSP machine. Firstly, the rotating pinless tool is moved over the groove to compact reinforcement particles and close the groove opening. This step is done to reduce the splashing out of reinforcement particles during processing. After compaction, the tool with a stirring pin is made to rotate at 1000 rpm and brought into contact with the copper plate. The generation of friction heat between the tool as well as the copper plate is attributed to the high-speed spinning of the tool. The heat generated by friction is sufficient to cause the copper material to undergo plasticization, while the presence of the stirring pin located at the bottom of the tool leads to significant deformation of the plasticized material. The processing tool effectively achieves transverse movement, resulting in a full mixing of copper and reinforced High Entropy Alloy (HEA) particles. Thus, the HEA particles were uniformly distributed in the tool-affected area. For achieving better mixing of reinforcement particles, the transverse speed of the processing tool is set at 30 mm/min^23^. The machine parameters were selected based on trial experiments conducted.

The developed composite is analyzed for its microstructure, hardness, and tensile characteristics. Cu HEA composite exposed fine grain structure and a maximum microhardness of 149 Hv is achieved for 15% HEA reinforcement. The introduction of HEA led to decrement in tensile properties but an increase in HEA % resulted in enhancement of tensile strength. Detailed discussion on the microstructure, hardness, and tensile behavior of the composite can be found in the author’s previous publication^32^.

**Figure 2:**
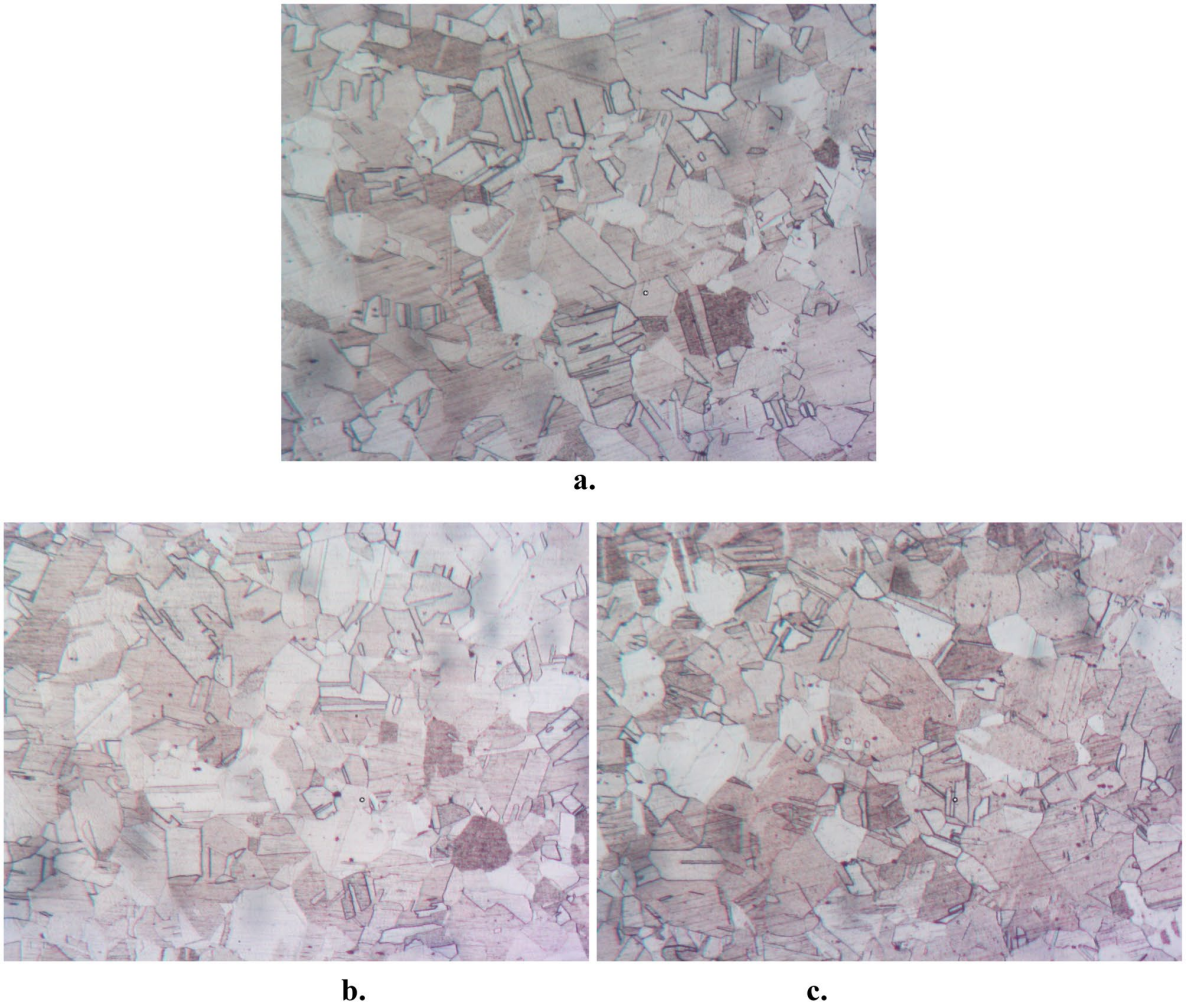
Microstructure of (**a**) base material, Cu with (**b**) 5 Vol. % HEA and (**c**) 15 Vol. % HEA.

Microstructure of the prepared material was observed employing an optical microscope and the results are provided in Fig. 2. Fine and equiaxed grain structures can be noted in stir zone as depicted in Fig. 2. The copper has a greater grain size than the HEA-reinforced copper, which can be attributed to both significant plastic deformation as well as heat created by friction amongst the tool and work undergoing FSP. This, in turn, leads to recrystallization. A comprehensive examination of the microstructure of the developed materials is extensively discussed in the author's prior research^32^.

### Wear analysis

The wear test is performed following the procedure outlined in the ASTM G99 standards. The test specimens used in the experiment were square-shaped with dimensions of 6 mm × 6 mm × 30 mm. These specimens were obtained by cutting from the core of the FSPed zone, as shown in Fig. 1b. The wear characteristics of the surface composite that was generated were evaluated by experimentation using the Pin-On-Disc equipment manufactured by Ducom, as depicted in Fig. 1c. The composite pin, which has been produced in advance, is designed to undergo frictional contact with a revolving disc composed of heat-treated EN31 steel possessing a hardness level of 65 HRC. Before each test, the pin and disc surface undergo a polishing process using an abrasive sheet to enhance the smoothness of the contact surface, hence facilitating improved contact. Following the polishing process, the components underwent a thorough cleansing procedure involving the use of acetone to eliminate any contaminants present on the contact surface. Subsequently, the components were subjected to a drying process to ensure the complete removal of any residual dirt particles. The assessment of the wear pattern of test composite is carried out with a track radius of 30 mm followed by the determination of the CoF as well as wear rate during the testing process. Wear rate is determined by measuring the mass of the test specimen both before and after the testing process. The determination of the coefficient of friction is achieved utilizing the data gathering system integrated inside the testing apparatus. The wear test was conducted to investigate the impact of significant variables, including sliding acceleration, load, and sliding length.

Table 2 presents input process parameters, their corresponding values, and the comprehensive experimentation conducted for all feasible combinations. The selection of the range for each parameter was determined by considering the capabilities of the machine and conducting trial experiments.

**Table 2:** Input process parameters and their levels.

Parameter	Level I	Level II	Level III	Level IV
Vol% HEA (V)	0%	5%	10%	15%
Sliding speed (S)	0.5 m/s	1 m/s	1.5 m/s	2 m/s
Load (L)	10 N	20 N	30 N	40 N
Sliding distance (D)	1000 m	2000 m	3000 m	4000 m

## Results and discussion

### Effect of control functions on wear rate

**Figure 3:**
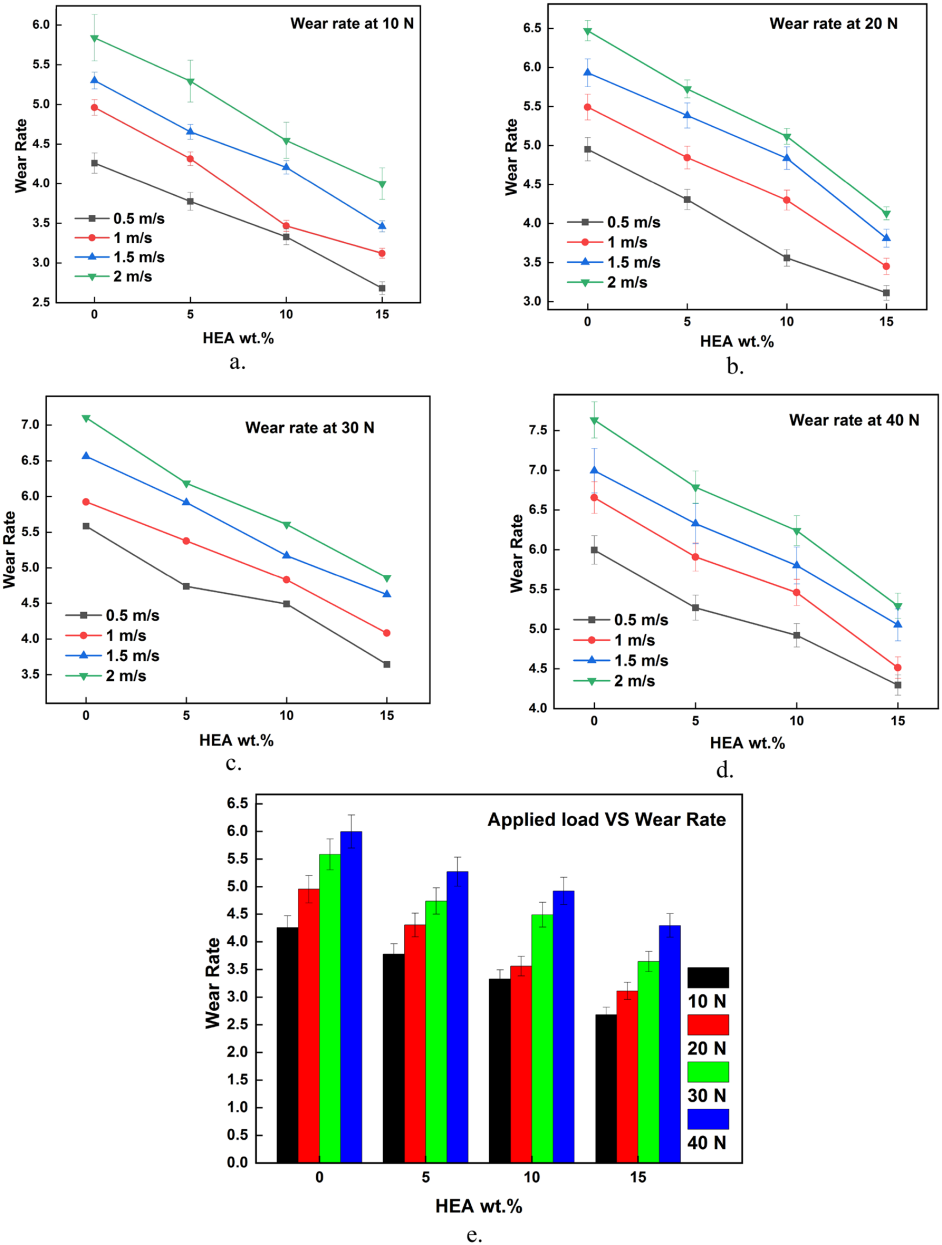
(**a**–**e**) Effects of load on WR at constant sliding distance.

**Figure 4:**
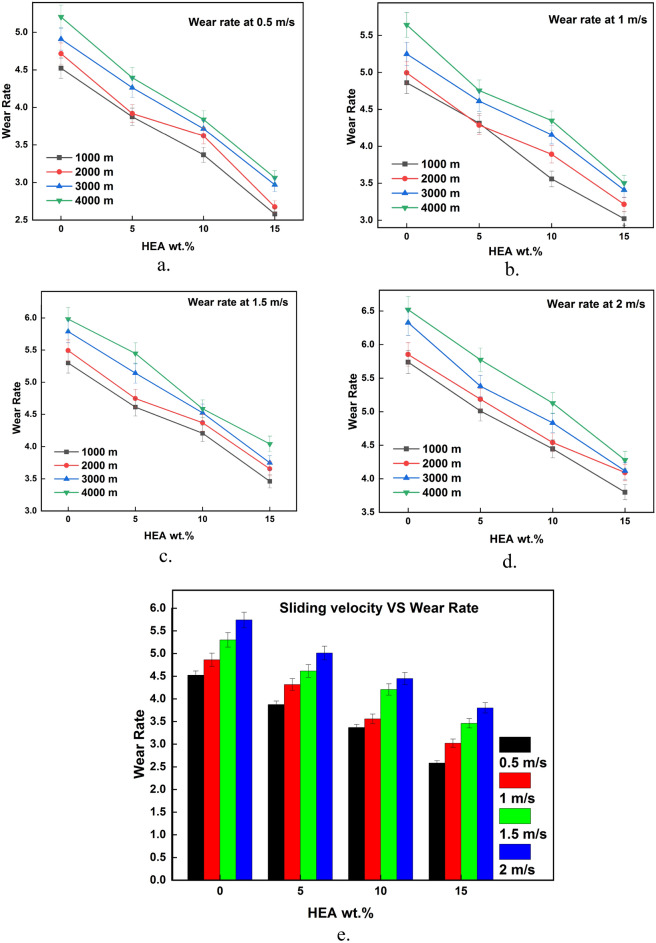
(**a**–**e**) Effects of sliding velocity on WR at constant load.

**Figure 5:**
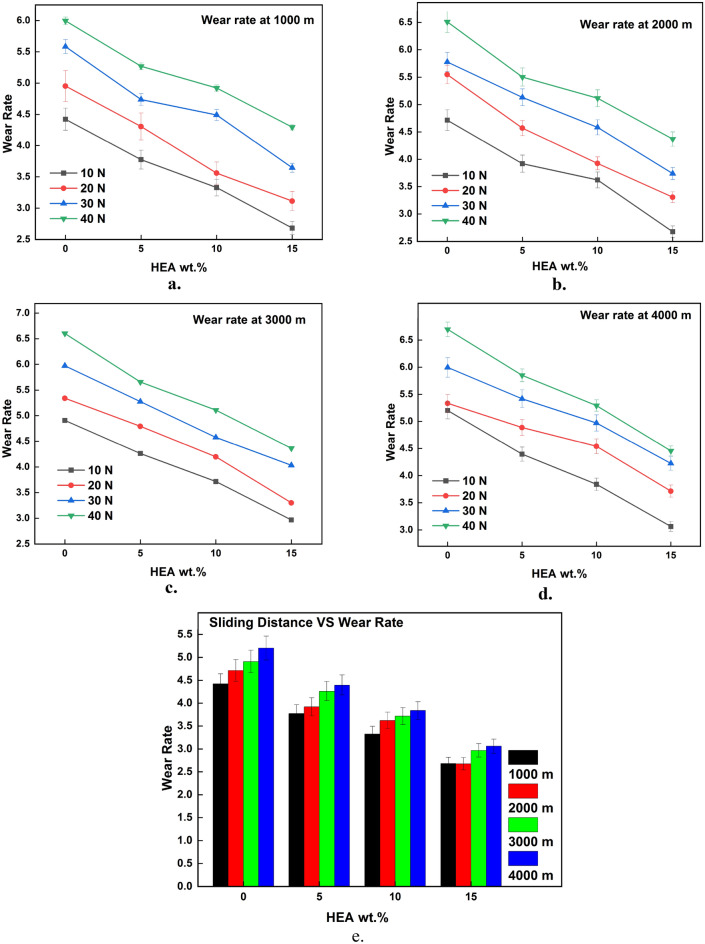
(**a**–**e**) Effects of sliding distance on WR at constant sliding velocity.

**Figure 6:**
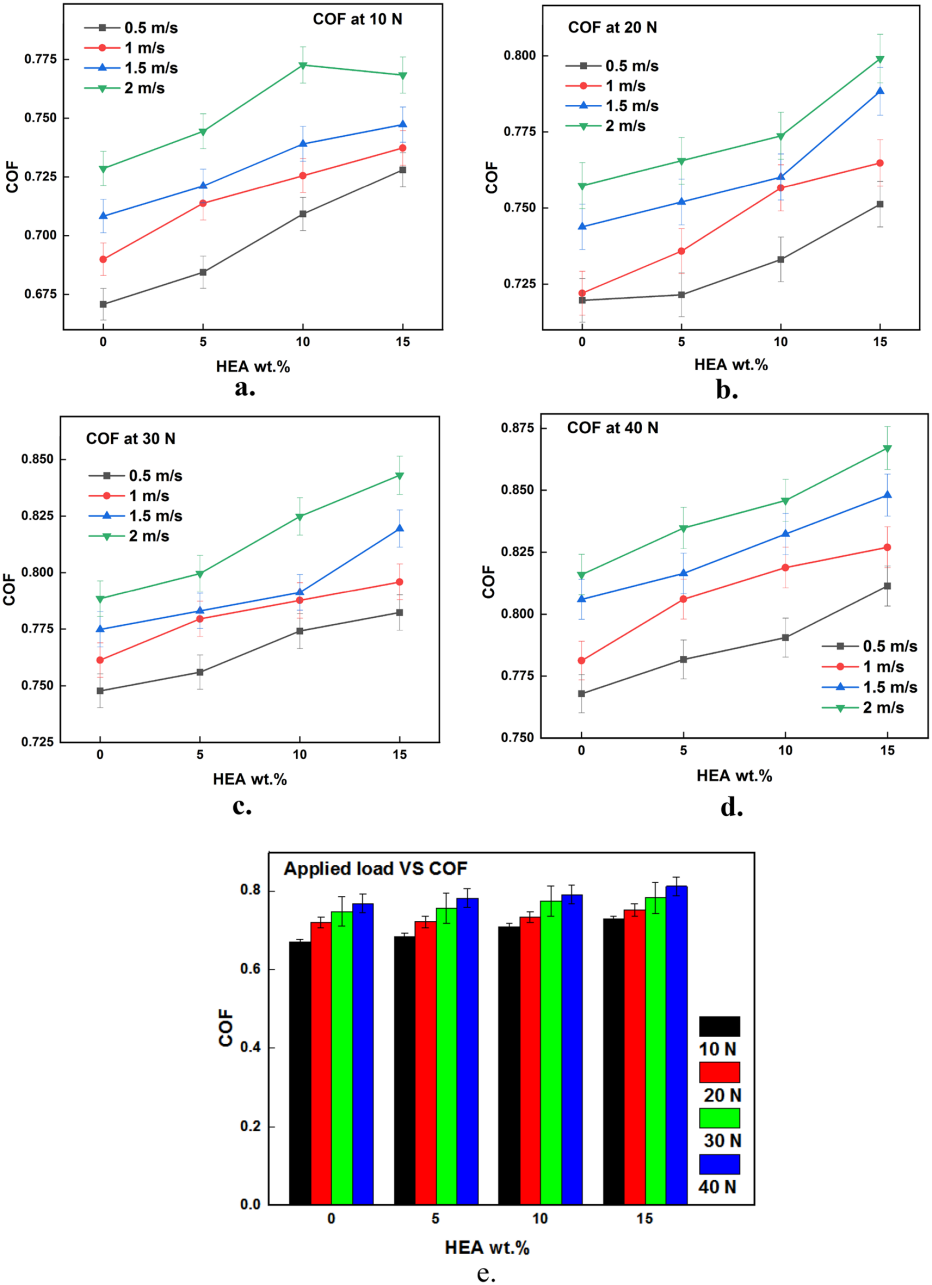
(**a**–**e**) Effects of load on CoF at constant sliding distance.

**Figure 7:**
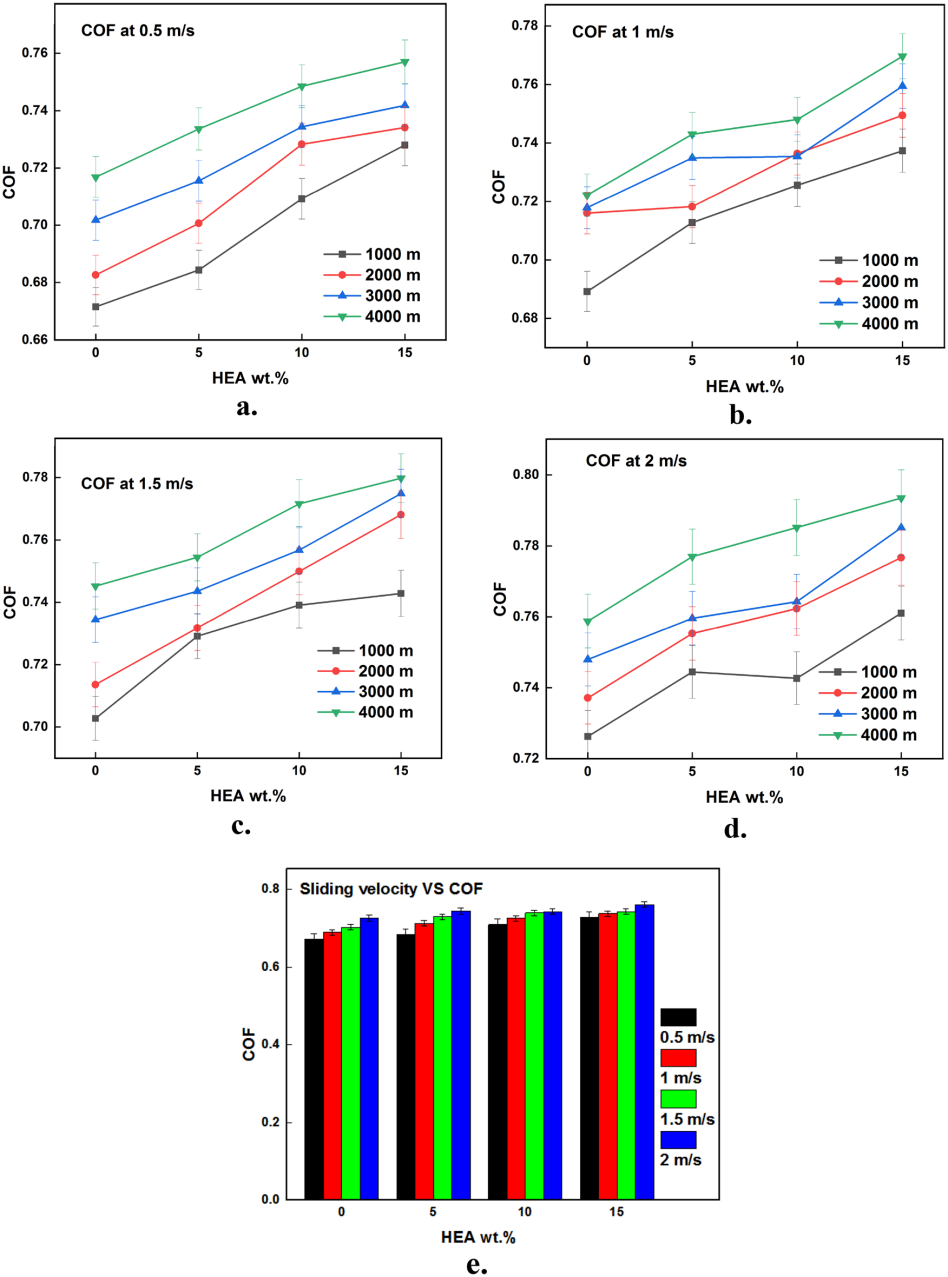
(**a**–**e**) Effects of sliding velocity on CoF at constant load.

**Figure 8:**
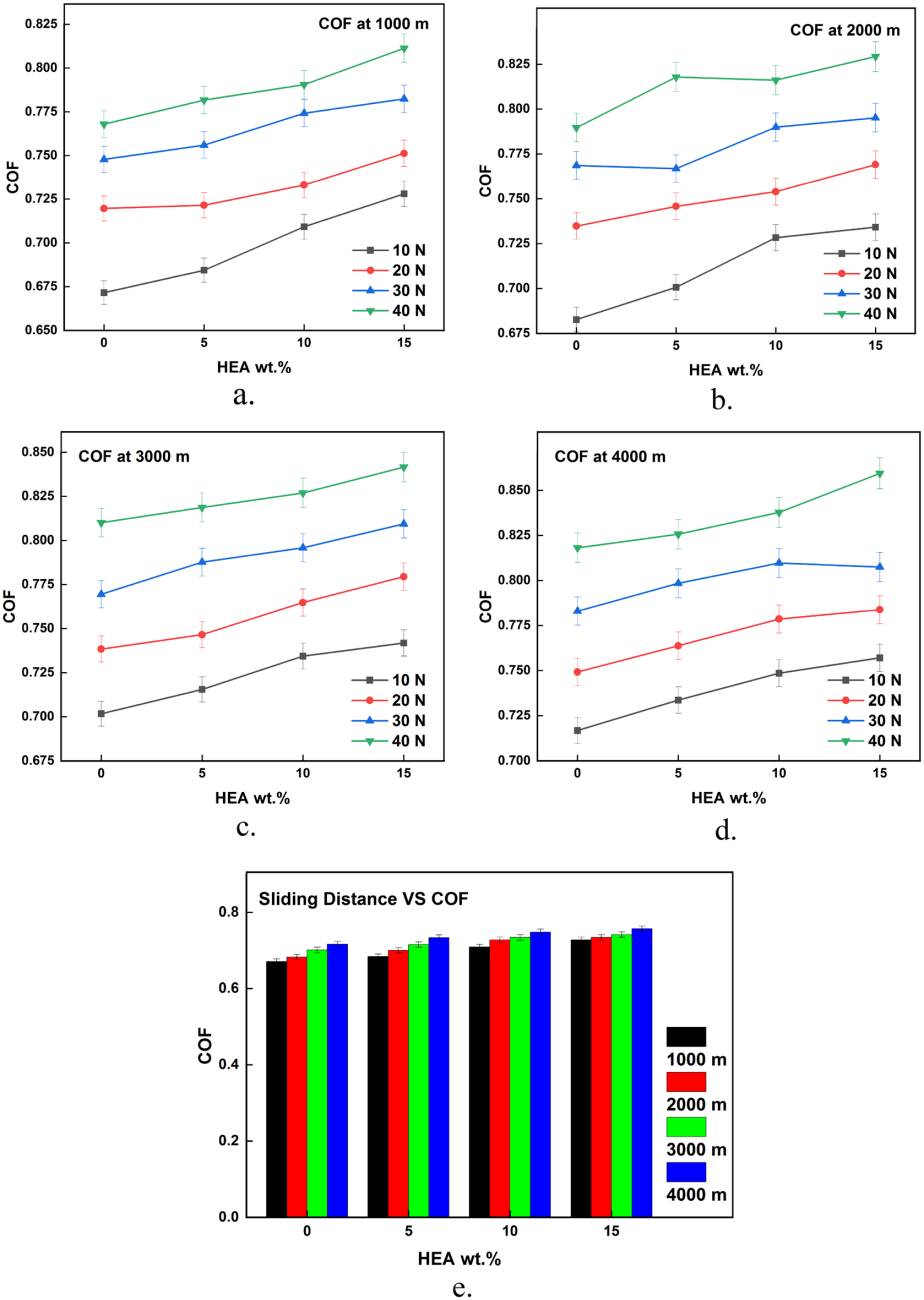
(**a**–**e**) Effect of sliding distance on CoF at constant sliding velocity.

The results of wear test acquired from the testing are plotted against the input parameters and the same is illustrated in Figs. 3, 4, 5, 6, 7, 8. It is very clear from the graphs shown in Figs. 3, 4, 5 that the reinforced HEA particles offer great influence on wear-resistant capability of copper matrix. The capability to resist the wear when sliding over the counterpart was momentously improved when the HEA particles were added and it improved further for every addition of the HEA reinforcing medium. The wear rate considerably declines on adding 5% HEA reinforcement with copper due to load carrying capability and increase in hardness of the copper with HEA addition^33^. It could be concluded that higher material hardness leads to raised wear resistance^34^. Therefore, the copper's resistance to wear is enhanced as the amount of high-entropy alloy (HEA) added increases, as this leads to an increase in the hardness of the copper. Moreover, the hardness of the copper matrix is enhanced due to the formation of a fine-grain structure resulting from the intense stirring action of the Friction Stir Processing (FSP) tool during the creation of test composite material^32^. Wear rate of copper exhibits a significant decrease when 10% high-entropy alloy (HEA) is used, indicating the enhanced wear resistance of the reinforced material. However, while the reduction in wear rate is notable, it is comparatively smaller for the composite reinforced with 15% HEA in comparison to the surface composite reinforced with 10% HEA.

The introduction of asperities due to the presence of reinforced HEA particles is also a major reason behind increased wear resistance^35^. When a reinforced material slides against a hard counterpart, soft matrix material in the vicinity of reinforcement material will be worn out. Such loss of material around hard reinforcement makes them exposed to hard counterparts i.e., asperities formation on the surface. So, the reinforced hard material directly comes into contact with the counter disc, and due to the hard nature of the reinforcements the material loss decreases i.e., the wear rate decreases. The production of asperities, as well as their size and spacing, is determined by various factors including the characteristics, quantity, dimensions, and arrangement of the reinforcing material. The formation of asperities is more pronounced in fine-sized particles compared to larger particles, and the uniform distribution of reinforcement is a crucial consideration in this context. The variation in distance between these asperities governs the surface roughness, which depends on the particle size of the reinforcement. Composites containing fine particles may possess less roughness compared to composites having coarse particles. Thus, fine particles arranged in the matrix having lesser interparticle distance will offer higher wear resistance over the coarse one^36^.

The sliding wear test demonstrates a significant correlation between the applied stress and the rate of wear, as illustrated in Fig. 3a–e. Wear rate of the Cu matrix and surface composites augmented with high-entropy alloys (HEAs) exhibits a significant rise as the applied load is increased. The figure demonstrates a discernible connection between the wear rate and load, indicating that the wear rate exhibits a positive correlation with increasing load increments. The observed correlation between the increasing load and the accelerated rate of wear in case of composite and copper matrix suggests a corresponding increase in material loss under higher loads. This increase in material loss is due to increased plastic deformation induced at the higher loading conditions during sliding^37^. This increase in plastic deformation results in material transfer between the sliding parts, from soft material to hard material which will come out as debris. This loss of material due to plastic deformation increases higher load and hence wear rate increases.

It is also a well-known fact that materials generally have thin films or layers of oxide on their surface. This oxide layer initially hinders the sliding parts from coming into direct contact which will be broken with ease when the load applied is increased. This removal of the oxide layer on the surface results in direct contact between the sliding parts. Higher contact pressure at the higher load results in fracture of the oxide layer that exposes the pin material to the hard counter disc which in turn induces plastic deformation. The exposed pin material welds together with the hard counter disc surface due to higher contact pressure and the welded material delaminates from the soft surface due to sliding action. The welded mixed material usually comes out as wear debris and sometimes it is transferred to hard material from the soft material surface as a film. This loss of material in the form of wear debris or transferred layer at the higher loading conditions results in increased wear loss. Hence, the rate of wear for the base copper and surface composites with HEA reinforcement increases at higher loads.

However, it is evident from the experimental findings presented in Fig. 3a–e that the rise in wear rate for the surface composites reinforced with high entropy alloys (HEA) is comparatively lower as the load increases, in contrast to the basic copper material. While the wear rate of composites does increase with an increase in load, the extent of this increase is comparatively smaller than the increase in wear rate observed for the basic matrix. This finding demonstrates the enhanced resistance to wear exhibited by the reinforced high-entropy alloy (HEA) at elevated loading conditions.

The impact of sliding speed over the rate of wear of the unreinforced and HEA-reinforced surface composite is depicted in Fig. 4a–e. The rate of wear increased considerably with a rise in sliding velocity during the wear test. At higher sliding velocities, the shear strain increases which results in increased material loss i.e., wear rate increases. Also, at higher sliding velocity and load, the temperature at the pin and disc interface increases which leads to thermal softening^38,39^. The microthermal softening that occurs at elevated sliding velocity ends in a reduction of bonding strength among the constituents of composite material^40^. This reduction in strength leads to particle detachment and more plastic deformation which tends to increase the wear rate.

Sliding distance has a significant effect on the wear rate as depicted in Fig. 5a–e. The rate of wear during the wear test increased considerably with an increase in sliding distance. The increase in rate of wear is high at the initial stages of sliding i.e., at a lesser sliding distance. This may be due to the form of wear at that stage which is abrasive. When the sliding distance increases the rate of wear also increases but the increase in wear rate is low when compared to the initial stages. This decrement or steadiness in the wear rate increment shows the end of the initial transition period i.e., run-in wear. So, the sudden increase in wear rate becomes steady once the sliding distance reaches a particular value^41^. Once the sliding distance is higher, the contact time between the sliding parts is also higher which results in increased temperature at the interface. When two surfaces are in sliding contact for more time, more heat energy will be produced at the interface as a result of friction. This increased frictional heat results in a temperature rise at the interface which softens the material. When the material becomes soft, it easily undergoes plastic deformation and there is a chance of reinforcement pulling out from the matrix. This increase in plastic deformation results in more material loss and hence wear rate^42^.

### Effect of input parameters on CoF

Figures 6, 7, 8 depicts the effects of HEA inclusion on the CoF throughout a dry sliding wear test, which contradicts its influence on the rate of wear. The incorporation of HEA to a copper matrix enhances the coefficient of friction while decreasing the wear rate. Figures 6, 7, 8 clearly show the effect of the considered input parameters on CoF. The increased CoF with HEA augmentation can be attributable to the reinforced HEAs' improved load-carrying performance. The matrix material, that is softer than the reinforcing phase, will wear out initially, allowing the harder reinforcement phase to protrude from the composite surface. Due to the hard nature of the sliding parts, the friction force increases when this projected hard reinforcement comes in direct contact with the counterpart^43^. Similarly, the increase in hardness of the copper with HEA addition is also attributed to the increase in CoF. It is owed to the fact that harder materials resist plastic deformation and hence the frictional force between the sliding parts increases.

The rise in CoF with a higher load applied during the wear test as illustrated in Fig. 6a–e is due to increased contact pressure at the interface. With the increase in load, the sliding parts were pressed against each other and it resulted in higher pressure at the interface. More resistance is offered by the counterparts during sliding owing to a higher pressure which results in higher friction at higher loads. Additionally, the soft matrix around the reinforcement easily wears out at higher loads which in turn results in contact between reinforcement protrusions and hard counterpart. This sliding of two harder phases results in a higher coefficient of friction^43^.

The CoF values increase considerably with an increase in sliding velocity as depicted in Fig. 7a–e. There is a chance of mechanically mixed layer formation at the sliding interface when parts are sliding at lower velocities^44^. The presence of a mechanically mixed layer serves to prevent direct contact between sliding components, resulting in the lower CoF. On the other hand, this mixed layer is sheared away when the sliding velocity is high. As a result, the pin material makes direct contact with the hard counter disc which causes higher frictional values. An increase in interface temperature at higher sliding velocities makes more asperity junctions which result in higher CoF^45^.

As the duration of sliding time rises, there is an observed increase in material loss. This leads to the protrusion of reinforced particles, resulting in contact with the harder counterpart. This contact between the hard surfaces increases CoF when sliding for more distance as shown in Fig. 8a–e. Also, it is a common fact that the relative motion among the sliding parts generates frictional heat at the interface. As the motion between the sliding parts is continuous for a long time at the higher sliding distances, the time for dissipating the generated frictional heat is almost nil. This increased temperature at the interface softens the material which sticks with its counterpart simply and makes the pin hard to slide i.e., frictional force increases. Increases in CoF with an increase in sliding distance may also be due to the protrusion of hard reinforcement particles from the matrix surface as a result of material loss at higher sliding distances^46^.

### Worn surface analysis

**Figure 9:**
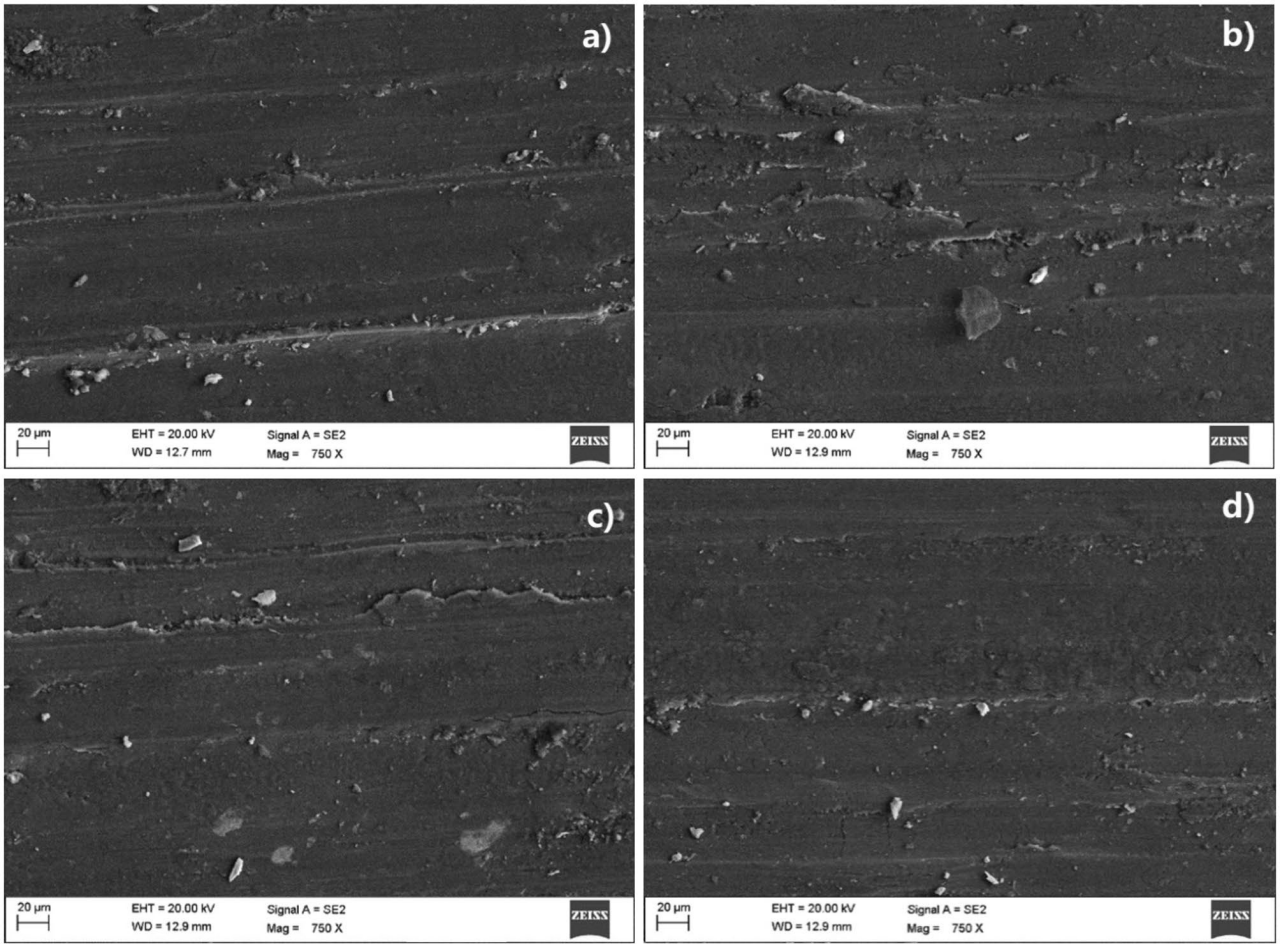
The worn-out surface of the (**a**) base material (**b**) Cu + 5% HEA, (**c**) Cu + 10% HEA, and (**d**) Cu + 15% HEA surface composites tested under mild conditions (L = 10 N, S = 0.5 m/s, D = 1000 m).

**Figure 10:**
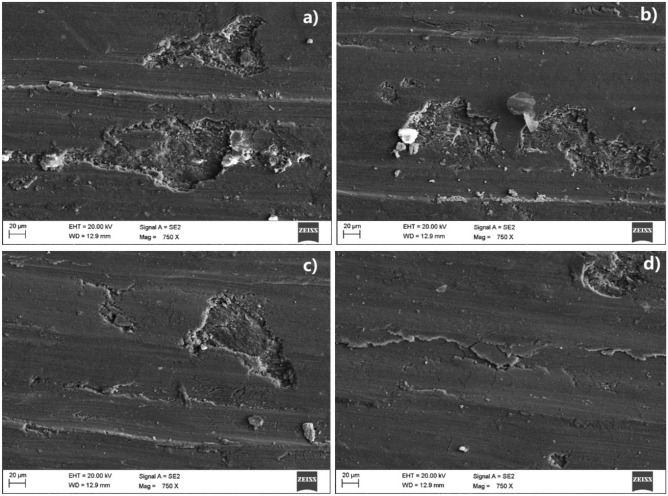
The worn-out surface of the (**a**) base material, (**b**) Cu + 5% HEA, (**c**) Cu + 10% HEA, and (**d**) Cu + 15% HEA surface composites tested under severe conditions (L = 40 N, S = 2 m/s, D = 4000 m).

The analysis of the worn surface of wear test specimens is essential for comprehending the wear characteristics exhibited by the material. Worn-out surface SEM images of the copper and the developed novel Cu-HEA composites tested under different sliding conditions are given in Figs. 9 and 10. It can be clearly understood from the SEM images that the surface damage during the wear test varies greatly with HEA % and sliding conditions. The developed copper surface composite with nil or lesser reinforcement content suffers exceedingly while less damage is encountered by the surface composite with more HEA inclusion.

The SEM micrographs presented in Fig. 9 provide empirical evidence supporting the improved wear resistance of copper when reinforced with HEA. The observed phenomenon could perhaps be attributed to the presence of strong interfacial bonding between the copper and the reinforced HEA^47^. The micrographs depicted in Fig. 9 illustrate the worn surfaces that occur in the copper matrix and composites with added high-entropy alloys (HEAs). These surfaces were subjected to light loading conditions, characterized by a minimal load, lower sliding velocity, and shorter distance. The level of surface damage experienced by the materials subjected to mild sliding conditions is minimal, as illustrated in Fig. 9. The copper matrix material exhibits deep and wide grooves, together with the presence of tiny cracks, as observed on the worn-out surface of the 5% HEA-reinforced composite. The existence of these grooves signifies a greater degree of material erosion, and the dimensions of the grooves diminish as the amount of HEA added increases.

Figure 10 presents the observed condition of the copper's worn surface and its surface composites when subjected to rigorous loading conditions, characterized by increased load, velocity, and distance. The unreinforced copper material has a worn surface characterized by significant subsurface damage, which can be attributed to increased plastic deformation as well as surface fracture. When a greater load is imposed, the contact pressure along the interface of the sliding components is elevated, resulting in an increase in adhesion across the pin and disc. The occurrence of cracks is facilitated by the heightened adhesive force in the direction of sliding^48–50^. Increased friction at the contact during extreme conditions leads to the displacement of particles, resulting in the formation of cavities, as illustrated in Fig. 9. This observation serves as evidence for heightened wear. The surface damage of the composite with increased HEA reinforcement was significantly impacted by all the materials evaluated under harsh conditions, as illustrated in Fig. 10. This observation demonstrates the enhanced resistance to wear exhibited by the reinforced high-entropy alloy (HEA) at elevated loading conditions.

## Conclusion

A novel copper-based surface composite with HEA as reinforcement is fabricated through friction stir processing. A detailed study of wear characteristics of the developed composites and copper is analyzed with applied load, sliding distance, and sliding velocity as input parameters. The outcomes derived from the study are as follows:

- Wear of Cu–HEA surface MMC decreases concerning the rise in reinforcement percentage while CoF increases to some extent.
- Wear resistance considerably increases with the addition of HEA reinforcement in Cu due to load-carrying capability and an increase in hardness. Minimum wear loss is observed for the 15% HEA-reinforced composite.
- The wear parameters such as load, sliding speed, and sliding distance possess a positive correlation with the wear rate and a negative correlation with a coefficient of friction.
- Applied load has a severe effect on wear rate and CoF when compared to other wear parameters considered (sliding velocity and sliding distance).
- Worn surface analysis confirms that the addition of HEA with Cu drastically reduces the severity of surface damage that commonly occurs during sliding wear tests.

## Data Availability

The data is available within the manuscript.
